# Bisulphide of Carbon as a Remedy for Headache

**Published:** 1869-04

**Authors:** 


Bisulphide of Carbon as a Remedy for Headache.—In a communication to the *British Medical Journal*, (June 13th),

Dr. Geo. Kennion, of Harrogate, calls attention to a very simple remedy in many kinds of headache, viz., Bisulphide of Carbon. He says:

"Its mode of application is no less simple. A small quantity of the solution, (about two drachms), is poured upon cotton-wool, with which a small, wide-mouthed, glass-stoppered bottle is half-filled. This, of course, absorbs the fluid; and, when the remedy has to be used, the mouth of the bottle is to be applied closely (so that none of the volatile vapor may escape) to the temple, or behind the ear, or as near as possible to the seat of pain; and so held, for from three to five or six minutes. After it has been applied for a minute or two, a sensation is felt as if several leeches were biting the part; and, after the lapse of two, three, or four minutes more, the smarting and pain become rather severe, but subside almost immediately after the removal of the bottle. It is very seldom that any redness of the skin is produced. The effect of this application, as I have said, is generally immediate. It may be applied, if necessary, three or four times in the day.

The class of headaches in which this remedy is chiefly useful, is that which may be grouped under the wide term of 'nervous'. Thus neuralgic headache, periodic headache, hysterical headache, and even many kinds of dyspeptic headache, are almost invariably relieved by it; and, although the relief of a symptom is a very different affair, of course, from the removal of its cause, yet no one who has witnessed, (and who of us has not seen?) the agony and distress occasioned by severe and repeated headache, but must rejoice in having the power of affording relief in so prompt and simple a manner.

As regards the *modus operandi* of this remedy, it is difficult, perhaps, to form a certain opinion; but I am disposed to attribute it to the sedative effect of the vapor of the bisulphide, absorbed through the skin, and acting upon the superficial nerves of the part to which it is applied. The remarks of M. Delpech, (*Annales d'Hygiene* Jan. 1863,) point out very clearly the remarkable prostration of the whole nervous system produced in workmen, who, in certain manufactures, are exposed to the vapor arising from the solution of the bisulphide of carbon; and we can readily understand that a somewhat similar effect, upon a small scale, may be produced by the application of this vapor to a limited portion of the surface."

